# The impact of cervical cytobrush sampling on cervico-vaginal immune parameters and microbiota relevant to HIV susceptibility

**DOI:** 10.1038/s41598-020-65544-6

**Published:** 2020-05-22

**Authors:** A. Mohammadi, S. Bagherichimeh, M. C. Perry, A. Fazel, E. Tevlin, S. Huibner, W. Tharao, B. Coburn, R. Kaul

**Affiliations:** 1 Department of Medicine, University of Toronto, Toronto, Canada; 2 Department of Immunology, University of Toronto, Toronto, Canada; 3 Women’s Health in Women’s Hands Community Health Center, Toronto, Canada; 4 Department of Laboratory Medicine and Pathobiology, University of Toronto, Toronto, Canada

**Keywords:** Inflammation, Virology

## Abstract

The immunology and microbiota of the female genital tract (FGT) are key determinants of HIV susceptibility. Cervical cytobrush sampling is a relatively non-invasive method permitting the longitudinal assessment of endocervical immune cells, but effects on FGT immunology are unknown. Blood, cervico-vaginal secretions and cervical cytobrushes were collected from sexually transmitted infection (STI)-free women at baseline and after either 6 hours or 48 hours. Endocervical immune cell subsets were assessed by flow cytometry, and pro-inflammatory cytokines by multiplex ELISA. The density of *Lactobacillus* species and key bacterial vaginosis-associated bacterial taxa were determined by qPCR. Paired changes were assessed before and after cytobrush sampling. After 6 hours there were significant increases in CD4 + T cell, antigen presenting cell (APC) and neutrophil numbers; APC elevations persisted at 48 hours, while neutrophil and CD4 + T cell numbers returned to baseline. In addition, pro-inflammatory cytokine levels were increased at 6 hours and returned to baseline by 48 hours. No significant changes were observed in the absolute abundance of *Lactobacillus* species or BV-associated bacteria at either time point. Overall, cytobrush sampling altered genital immune parameters at 6 hours, but only APC number increases persisted at 48 hours. This should be considered in longitudinal analyses of FGT immunology.

## Introduction

Women account for 51% of people living with HIV worldwide, with most infections acquired during heterosexual intercourse^1^. HIV is acquired when the virus crosses the mucosal lining of the female genital tract (FGT) or rectum during sex, and the mucosal immune milieu at the time of HIV exposure is a critical determinant of outcome. Effective mucosal immune defenses mean that most HIV exposures do not result in productive infection, but elevated genital pro-inflammatory cytokine levels are directly linked with increased HIV acquisition^2,3^, likely due to epithelial barrier disruption and the recruitment of more HIV target cells^4^. Key mucosal HIV target cells are activated (HLA-DR+) CD4+ T cells expressing the HIV coreceptor CCR5, in particular Th17 cells (CCR6+)^5–7^. Furthermore, expression of the α4β7 mucosal homing integrin by blood CD4+ T cells not only homes HIV target cells to mucosal surfaces, but may also directly enhance HIV infection^8–10^. Virus dissemination after mucosal virus infection is also facilitated by mucosal Antigen Presenting Cells (APCs), including CD14- and CD14+ dendritic cells (DCs), which are essential for HIV infection of T cells *in trans*^11–13^.

The immune milieu of the female genital mucosa is substantially influenced by the adjacent genital microbiota^3,14^. Specifically, a low vaginal abundance of *Lactobacillus* species and a high relative abundance of anaerobic genera including *Gardnerella*, *Prevotella*, *Atopobium* and others, defined as molecular bacterial vaginosis (BV)^15^, increases genital inflammation and HIV acquisition^3,14,16,17^. The innate host response to these bacteria induces secretion of inflammatory cytokines such as IL-6 and IL-8, recruitment of HIV target cells and reduction of epithelial integrity^14,16,18–20^.

The interplay between genital immunology, microbiota and HIV transmission means that longitudinal studies with repeat genital sampling may help to define new clinical approaches for HIV prevention, but mucosal sampling itself may have important impacts on subsequent mucosal immune parameters. Commonly used methods of genital sampling include endocervical cytobrush collection, ectocervical biopsy, or genital secretion collection via cervicovaginal lavage, swab or SoftCup; however, only the former two are suitable for cell sampling^21^. Ectocervical biopsies heal macroscopically within 5 days, with no significant increase in HIV target cells (CCR5 + CD4 + T cells) two weeks after sample collection, but sigmoid biopsy causes transient inflammation that persists in the peripheral blood for at least a week^22–24^. Cervical cytobrushes are a relatively non-invasive method to study endocervical immune cell populations and can be collected in a longitudinal fashion. However, the immune impact of endocervical cytobrush sampling is not well defined, and the nature and timing of potential alterations may be important for the planning of longitudinal studies of FGT immunology.

We hypothesized that cytobrush collection would induce transient genital immune alterations at 6 hours that would resolve by 48 hours. Our co-primary endpoints were levels of the inflammatory cytokines IL-1α and IL-8. Secondary endpoints were the number of neutrophils, number and proportion of APCs and CD4 + T cell subsets including Th17 cells and activated T cells.

## Results

### Participant characteristics

Twenty-two women were recruited, through Women’s Health in Women’s Hands Community Health Center (WHIWH) in Toronto, Canada. Participants had a median age of 25 years; demographic data are shown in Table 1. Participants were non-pregnant, had no symptomatic genital discharge, and screened negative for HIV and endocervical *N. gonorrhoeae* and *C. trachomatis* infection. Endocervical cytobrush sampling was performed at baseline for all 22 participants, and repeat sampling performed at either 6 hours (N = 11 participants) or 48 hours (N = 11 participants).

**Table 1:** Participant characteristics (N = 22).

Characteristic	Re-sampled at 6 hours (N = 11)	Re-sampled at 48 hours (N = 11)	Total (n = 22) no. (%)
**Age (median; range)**	26 (23–38)	24 (18–38)	25 (18–38)
Ethnicity			
Asian (East or South Asian)	2	6	8
African, Caribbean and Black (ACB)	3	1	4
Middle Eastern	2	1	3
Mixed	2	0	2
White	1	3	4
Latin American	1	0	1
**Contraceptive method**			
Oral contraceptive pill	7	5	12
Hormonal IUD	1	0	1
Hormonal patch	1	0	1
Copper IUD	1	1	2
Condom	3	6	9
None	1	1	2
**Vaginal douching**	3	0	3 (14%)
**Smoking**	1	0	1 (5%)
**Nugent-BV**	1	2	3 (14%)

### The impact of cytobrush sampling on CD4+ T cell subsets in the endocervix

**Figure 1:**
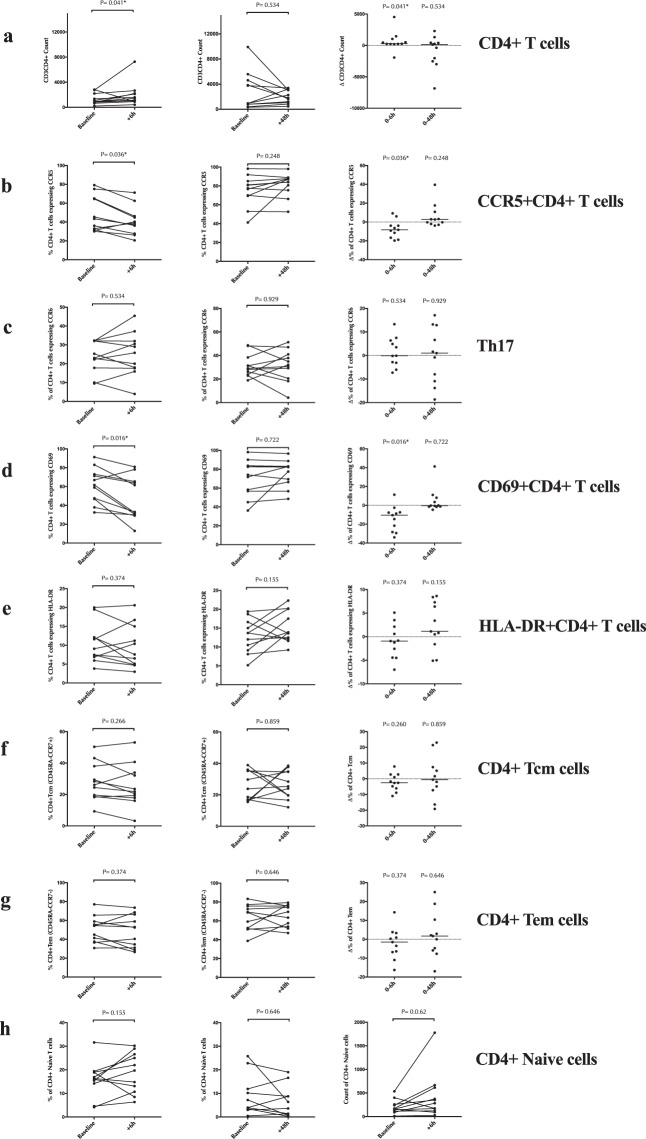
The effect of cytobrush sampling on endocervical CD4+ T cell counts and subsets. **(a)** Endocervical CD3CD4+ T cells count **(b)** Percentage of endocervical CD4+ T cells expressing CCR5 **(c)** Percentage of endocervical CD4 + T cells expressing CCR6 **(d)** Percentage of endocervical CD4+ T cells expressing CD69 **(e)** Percentage of endocervical CD4+ T cells expressing HLA-DR **(f)** Percentage of endocervical CD4+ central memory T cells (Tcm: CD45RA-CCR7+) **(g)** Percentage of endocervical CD4+ effector memory T cells (Tem: CD45RA-CCR7−) **(h)** Percentage of endocervical CD4 + naive T cells (CD45RA + CCR7+), 6 hours and 48 hours after cytobrush sampling. Statistical comparisons were performed using Wilcoxon Signed Ranked test.

Given that inflammation in the FGT can recruit HIV target cells, we first examined the impact of endocervical sampling on the number of CD4+ T cells per cytobrush, and the proportion/number of endocervical CCR5 + CD4+ T cells, Th17 Cells (CCR6+) and activated CD4+ T cells (HLA-DR + ; see supplementary Fig. 1a for gating strategy). The total number of endocervical CD4+ T cells increased 6 hours after cytobrush sampling (median difference = +268, p = 0.041; Fig. 1a); there was no change in the number/proportion of endocervical Th17 cells, activated CD4+ T cells or memory CD4+ T cell subsets (all p > 0.1; Fig. 1c,e,f,g respectively), and the proportion of endocervical CD4+ T cells expressing CCR5 and early activation marker (CD69) actually decreased 6 hours after cytobrush sampling (median difference = −8.3, p = 0.036; median difference = −10.4, p = 0.016; Fig. 1b and d respectively). These endocervical T cell alterations were not detectable at 48 hours (all p > 0.1; Fig. 1). Cervical cytobrush collection was not associated with any changes in T cell parameters in the peripheral blood at any timepoint, specifically the proportion of CD4+ T cells expressing the HIV co-receptor CCR5, the mucosal homing integrin α4β7+ , or the activation marker (HLA-DR) (all p > 0.1).

### The impact of cytobrush collection on endocervical neutrophils

**Figure 2:**
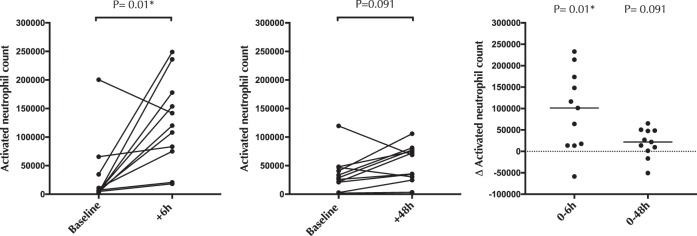
The effect of cytobrush sampling on endocervical activated neutrophils. Endocervical activated neutrophil counts 6 hours and 48 hours after cytobrush sampling. Statistical comparisons were performed using Wilcoxon Signed Ranked test.

Neutrophils are among the first immune cells to be recruited during tissue repair, and so we hypothesized that there would be an early influx of neutrophils into the endocervical mucosa after cytobrush collection. Activated endocervical neutrophils were defined as CD3-CD19-CD14-CD16 + CD15+ cells expressing CD66b (see supplementary Fig. 1b for gating strategy). The number of endocervical activated neutrophils increased significantly 6 hours after cytobrush collection (median difference = +101,313, p = 0.010; Fig. 2). Again, these changes were not detected at 48 hours (median difference = +21,832, p = 0.091; Fig. 2).

### Cytobrush collection impact on endocervical Antigen Presenting Cells (APCs)

**Figure 3:**
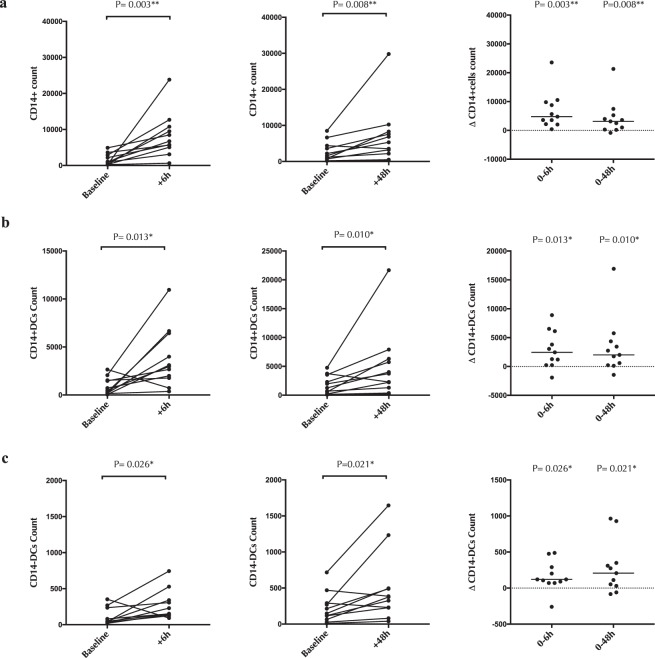
The effect of cytobrush sampling on endocervical monocyte/macrophage and DC numbers. Counts of **(a)** endocervical macrophages/monocytes (CD14+ cells) **(b)** endocervical CD14+ DCs and **(c)** endocervical CD14-DCs, 6 hours and 48 hours after cytobrush sampling. Statistical comparisons were performed using Wilcoxon Signed Ranked test.

**Figure 4:**
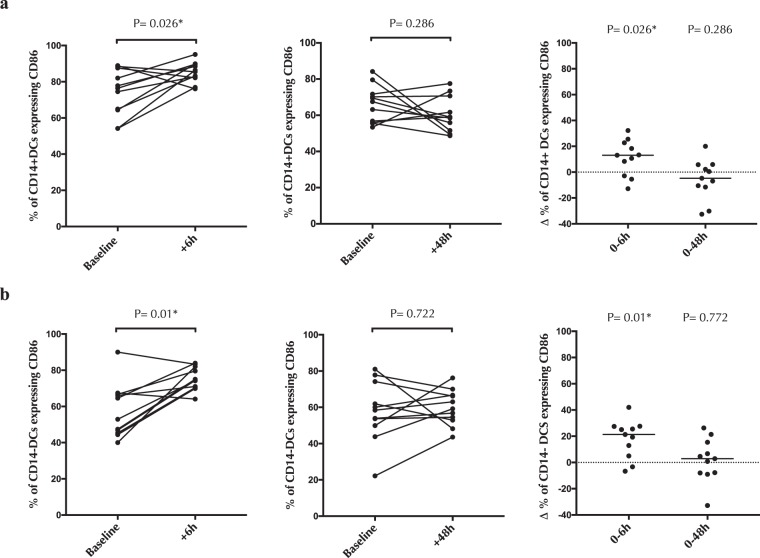
The effect of cytobrush sampling on endocervical DC activation. Percentage of **(a)** endocervical CD14+ DCs and **(b)** endocervical CD14-DCs expressing activation marker (CD86) 6 hours and 48 hours after cytobrush sampling. Statistical comparisons were performed using Wilcoxon Signed Ranked test.

Next, we assessed changes in endocervical antigen presenting cells after cytobrush sampling by focusing on monocytes/macrophages (CD14+ cells) and DC subsets (both CD14 + and CD14-DCs) (see supplementary Figure 1c and d for gating strategy). Cytobrush sampling induced an increase in the number of endocervical CD14+ monocytes and both DC subsets (CD14 + DCs and CD14-DCs) that was apparent at both 6 hours (median difference = +4,773, p = 0.003; +2455, p = 0.013; +121, p = 0.026 respectively; Fig. 3a,b,c respectively) and 48 hours post-collection (median difference = +3,114, p = 0.008; +2,013, p = 0.010; +207, p = 0.021 respectively; Fig. 3a,b,c respectively). Activation status of endocervical APCs was defined by the proportion of these cells expressing the activation marker CD86. Both DC subsets were more activated 6 hours but not 48 hours after sample collection (p < 0.03 and p > 0.2 respectively; Fig. 4a,b).

### The impact of cytobrush collection on cervico-vaginal cytokine levels

**Figure 5:**
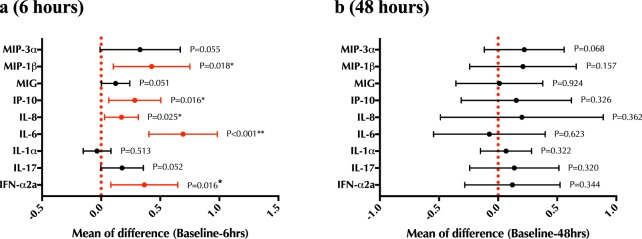
The effect of cytobrush sampling on cervico-vaginal cytokine concentrations. Change in concentration of undiluted cervico-vaginal cytokines from baseline to **(a)** 6 hours after cytobrush sampling, and **(b)** 48 hours after cytobrush sampling. Cytokine values were log10 transformed, and statistical comparisons were performed using a paired t-test.

The impact of cytobrush sampling on levels of pro-inflammatory cytokines (IL-1α, IL-6) and chemokines important for HIV acquisition (IL-8, MIP-3α, MIP-1β, MIG and IP-10) was then assessed. At baseline (pre-sampling), there was no significant difference in any genital cytokine levels between the two participant groups (ie: those re-sampled at 6 hours or 48 hours; all p > 0.5; Supplementary Fig. 2). Levels of IL-6, IL-8, IP-10, MIP-1β, IFNα2a increased 6 hours after sampling (all p < 0.05), with no impact on other cytokine/chemokine levels (all p > 0.05; Fig. 5a). There was no significant increase in any cytokines/chemokines 48 hours after cytobrush sampling (all p > 0.06; Fig. 5b).

### Impact of cytobrush collection on the genital microbiota

**Figure 6:**
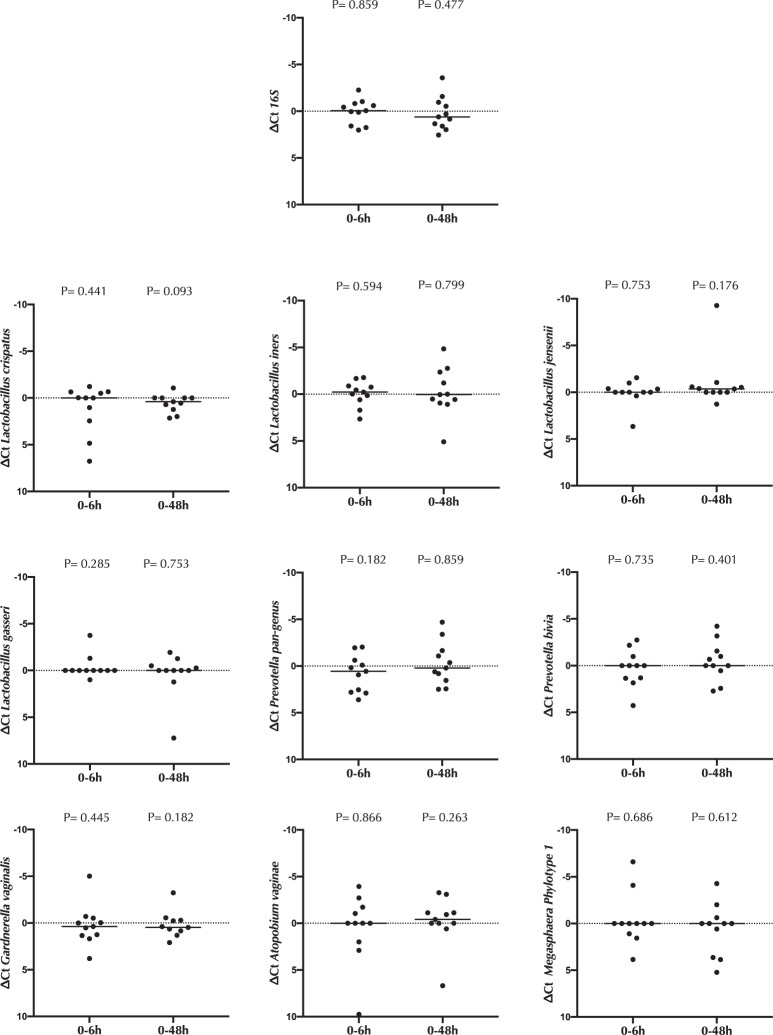
Impact of cytobrush sampling on the cervico-vaginal microbiota. The change (Δ) in the absolute abundance of 9 key cervico-vaginal bacterial species/taxa 6 hours (0-6 h) and 48 hours (0–48 h) after cytobrush sampling. Statistical comparisons were performed using Wilcoxon Signed Ranked test.

The absolute abundance of nine bacterial species/taxa that play a key role in the vaginal microbiota (*L. iners, L. gasseri, L. jensenii, L. crispatus, Prevotella spp, P. bivia, G. vaginalis, A. vaginae and Megasphaera phylotype 1*) was assessed at each study visit. Endocervical cytobrush sampling was not associated with changes in the absolute abundance of any bacterial species/taxa (Fig. 6).

## Discussion

The genital immune milieu and genital microbiota both play a key role in determining whether a sexual HIV exposure results in persistent viral infection or in virus repulsion^2–4,14,16^. For this reason, the longitudinal assessment of these mucosal parameters may be an important tool to test novel interventions that aim to reduce HIV risk. Cervical cytobrushes are frequently used as a relatively rapid and non-invasive method to assess endocervical immune cell populations^21^. However, the effect of cytobrush sampling on FGT immunology and microbiota composition has not been assessed, and this may be important to define given the impact of gut mucosal biopsies on systemic immune parameters^24^. Our study demonstrates that cervical cytobrush sampling substantially alters genital cytokines and several endocervical immune cell subsets (T cells, neutrophils and dendritic cells) within a few hours of sampling, without affecting the local microbiota. The majority of these immune changes had resolved within 48 hours, although dendritic cell numbers remained elevated. This has important implications for the planning, performance and interpretation of translational research studies that involve longitudinal assessment of genital immune parameters.

Longitudinal genital immune studies often focus on mucosal CD4+ T cell numbers and subsets, since these are key HIV target cells. Particularly relevant virus targets are thought to be activated CD4+ T cells, and CD4 + T cells that express the HIV co-receptor CCR5 and/or the Th17 lineage marker CCR6^5–7^. In addition, expression of the mucosal homing integrin α4β7 may enhance HIV susceptibility, and the frequency of α4β7 + CD4+ T cells in blood has been directly correlated with HIV acquisition^9,10^. In contrast to immune changes induced by a sigmoid biopsy, we saw no change in blood CD4+ T cell subsets, including α4β7 expression, after cervical cytobrush collection. While there was a modest but significant transient increase in the number of endocervical CD4+ T cells shortly after cytobrush collection, we believe that this was likely due to blood contamination since these CD4+ T cells expressed significantly less CCR5 and lower levels of CD69, which is typical of blood (rather than mucosal) T cells^25^.

The post-sampling increases that we observe in the number of activated endocervical neutrophils and APCs, the former being transient and the latter more sustained, are in keeping with local recruitment, and fit with what we know about epithelial injury and healing^26^. Specifically, since the neutrophil chemoattractant IL-8 is expressed by damaged epithelial cells and is associated with neutrophil recruitment, a rapid endocervical influx of neutrophils after cytobrush collection would be expected^27^. Epithelial healing is then associated with a later and more prolonged influx of monocytes and APCs that are activated by the microbiota, produce IL-6 and enhance healing through induction of regenerative responses by epithelial progenitor cells^26,28,29^; this is in keeping with the concurrent increase in IL-8 and IL-6 that we observed. Blood contamination is only likely responsible for a minority of the post-sampling cervical neutrophil increase, since in blood these cells express much lower levels of the activation marker CD66b than was observed. Overall, the immune shifts that we observed suggest that epithelial injury by cytobrush sampling induced expression of pro-inflammatory cytokines and the recruitment of neutrophils and APCs to promote healing.

A genital microbiota that is enriched for diverse gram negative and positive anaerobes, with a reduced abundance of lactobacilli, is defined as molecular BV^15^ and has been clearly associated with increased pro-inflammatory cytokines, reduced epithelial barrier integrity and increased HIV risk^3,14,20^. However, it is not clear whether genital dysbiosis drives epithelial disruption, or whether a disrupted epithelium promotes colonization by BV-associated bacteria. Our study suggests the former direction of causation, since the direct induction of epithelial disruption by a cytobrush was not associated with alterations in the absolute abundance of either key lactobacilli or BV-associated bacterial species, although it is possible that this might not be the case for multiple sampling over time.

Despite demonstrating a clear impact of cytobrush sampling on mucosal immune parameters in the absence of microbiota alteration, our study does have several limitations. First, the relatively small sample size limits our power to detect significant changes in some cell subsets, such as neutrophil infiltration at 48 hours. In addition, longer follow up would be needed to define the time required for resolution of immune alterations that persisted at 48 hours, such as increased mucosal APC numbers. Our analysis of the vaginal microbiota assayed the abundance of key bacterial taxa and/or species using qPCR, which has the advantage of most accurately quantifying bacterial abundance; we can therefore be confident that there was no alteration in key bacterial taxa, but were not able to assess bacterial diversity as comprehensively as an unbiased approach such as 16S rRNA gene or metagenomic sequencing.

In conclusion, we demonstrate that endocervical cytobrush collection caused clear and significant short term alterations in several genital cell populations and cytokines, without altering the abundance of key bacterial taxa, but that most of these immune alterations (with the exception of increased APC numbers) had resolved within 48 hours. These findings are of particular importance to researchers planning longitudinal studies of genital immunology and HIV risk, and also suggest that a period of post-cytobrush sexual abstinence may be advisable in high risk participants such as female sex workers.

## Methods

### Participant enrollment and exclusion criteria

Women were recruited into this prospective cohort study through Women’s Health in Women’s Hands Community Health Center (WHIWH) in Toronto. The protocol was approved by the HIV Research Ethics Board at the University of Toronto. All research was performed in accordance with relevant guidelines and regulations. Flyers were posted within the WHIWH centre and across the University of Toronto St. George campus. The research nurses at WHIWH provided the detailed information about the study to potential participants. At the screening visit informed consent was taken from all participants and they were tested for sexually transmitted infections and pregnancy. Exclusion criteria were infection with HIV1/2, syphilis, *Neisseria gonorrhoeae* (GC) and/or *Chlamydia trachomatis* (CT); Ag <16 yrs; pregnancy; any genital ulcers or discharge; irregular bleeding; taking immunosuppressive medications and having taken antibiotics within one month prior to study enrollment.

### Study protocol and sampling

The study protocol consisted of three visits; screening, baseline and follow up visits. At the screening visit, blood and urine were collected for STI diagnostics. Eligible participants completed a demographic/behavioral questionnaire at the baseline visit and samples were collected, with repeat sampling after 6 hours or 48 hours. Samples were collected in the following order: SoftCup self-collection, study nurse vaginal swab collection, study nurse endocervical cytobrush collection, and blood collection. Cervico-vaginal secretions were self-collected using an Instead SoftCup (Evofem, San Diego, CA) inserted for 1 min and were used for cytokine and microbiota analysis. Two vaginal swabs, two endocervical cytobrushes and blood were collected by the study nurse. Each cytobrush was gently inserted into the cervical os, rotated through 360°, placed into R10 medium (RPMI 1640 with 10% heat-inactivated FBS [Sigma-Aldrich, Carlsbad, CA], 100 mg/ml streptomycin, 100 U/ml penicillin, and 13 GlutaMAX-1 [Life Technologies, Grand Island, NY] media) at 4 °C and transported to the laboratory within 30 min of collection.

The two cytobrushes were processed together, the combined cells were filtered through a 100-μm filter, washed, and divided into two equal aliquots for staining. Peripheral blood mononuclear cells (PBMCs) were isolated by Ficoll-Hypaque density centrifugation at 1500 rpm for 30 min, counted, and washed in R10 medium. One aliquot of one million PBMCs was used for staining of T cell subsets.

### STI and BV diagnosis

A vaginal swab was smeared onto a glass slide, air-dried and Gram’s stained to diagnose bacterial vaginosis (BV) using Nugent criteria and to screen for vaginal yeast. Testing for GC and CT were done on first-void urine at Mount Sinai hospital by nucleic acid amplification test (NAAT; ProbeTech Assay, BD, Sparks, MD). Testing for HIV1/2 and syphilis were performed by chemiluminescent microparticle immunoassay (CMIA) (ARCHITECT System, Abbott GmbH & Co. KG).

### Immune cell phenotyping

Endocervical cells and PBMCs were stained with two panels of Abs to characterize various T cell subsets and neutrophils/APC populations. The T cell panel consisted of CD45RA-FITC (BioLegend), CD8- Percp cy5.5 (eBioscience), β7-APC (BD Biosciences), CD127-APCef780 (eBioscience), CD25-BV421(BD Biosciences), CD4-BV650 (BD Biosciences), CCR6-BV711 (BD Biosciences), CD3-BV785 (BD Biosciences), α4-PE (BD Biosciences), CCR5-PE-CF549(BD Biosciences), CCR7-Pe-cy7 (BD Biosciences), HLA-DR-BUV395 (BD Biosciences), CD69-BUV737 (BD Biosciences) and Live/Dead Aqua (Invitrogen). The neutrophils/APCs panel consisted of CD14-FITC (BioLegend), CD66b-percp-cy5.5 (BioLegend), BDCA-2-APC (BioLegend), CD45-APC-fire (BioLegend), CD16-BV421 (BD Biosciences), CD83-BV650 (BD Biosciences), CD11c-BV785 (BD Biosciences), CD15-Pe (BD Biosciences), CD123- PECF549 (BD Biosciences), CD86-Pe-cy7 (BD Biosciences), HLA-DR- BUV395 (BD Biosciences), CD3/CD19- BUV-737 (BD Biosciences) and Live/ Dead Aqua (Invitrogen). Cells were enumerated using a BD LSR Fortessa X20 flow cytometer (BD Systems) and analyzed with FlowJo 10.4.1 software (TreeStar, Ashland, OR) by the same researcher for consistency. Fluorescence minus one (FMO) and isotype controls were used to establish proper gating.

For each cytobrush sample, all isolated endocervical cells were run through the cytometer, allowing for the endocervical immune cell populations to be quantified as the total number of cells/cytobrush.

### Cytokine analysis

Cervico-vaginal secretions (CVS) collected by SoftCup as described previously (see method) were diluted 10-fold using sterile PBS and spun down at 1730g for 10 min. Subsequently, the supernatant was frozen at −80 °C for cytokine analysis. The levels of cytokines IL-1α, IP-10, IL-8, MIP-3α, MIP-1β, IL-17a, IFN-α2a, IL-6, and MIG were measured in duplicate by Multiplex ELISA according to the protocol (Meso Scale Discovery, Rockville, MD). The supernatant of cervical secretion was plated at 25 μl per well. The standard curve was used to determine the lower and upper limit of detection and concentration of each analyte (pg/ml). Any sample above the upper level of detection was diluted and the multiplex ELISA was repeated. The LLODs were as follow: IFNα2a = 4.82 pg/ml; IL-17 = 8.3 pg/ml; MIP-3α= 2.7 pg/ml; IL-6 = 1.27 pg/ml; IL-1α= 99.6 pg/ml; IL-8 = 1.24 pg/ml; MIG = 2.47 pg/ml; IP-10 = 42.1 pg/ml; MIP-1β= 11.5 pg/ml. Samples that were below the limit of detection (LLOD) were given the LLOD value. Samples that were above the LLOD with a CV repeatedly higher than 30 were excluded from analysis. All samples were run by a researcher blinded to the status of participants. CVS samples provided at both study visits were assessed on the same plate to account for the plate-to-plate variability.

### DNA Isolation, bacterial load estimation and data analysis

250 μl of the pellet obtained from the CVS was used to isolate DNA using the Qiagen DNEasy PowerSoil kit (Qiagen), according to the manufacturer’s instructions. DNA was eluted in 100 μl of the Qiagen elution buffer.

Overall bacterial densities were measured using a universal 16S quantitative polymerase chain reaction adopted from Nadkarni *et al*.^30^. Total Prevotella spp. were quantified using a quantitative polymerase chain reaction assay adopted from Martin *et al*.^31^. *Prevotella bivia* was quantified using primers designed using the pipeline outlined in Schneeberger *et al*.^32^. *Lactobacilli spp. L. iners, L. gasseri, L. jensenii* and *L. crispatus* were quantified using a multiplex quantitative polymerase chain reaction assay adopted from Balashov *et al*.^33^. *Atopobium vaginae, Gardnerella vaginalis* and *Megasphaera* phylotype 1 were quantified using a multiplex quantitative polymerase chain reaction adopted from J. G. Kusters *et al*.^34^. Primer and probe sequences and assay concentrations are outlined in supplementary Table 1S.

Amplification and detection by real-time PCR were performed with the QuantStudio 6 Flex Real-Time PCR System (Thermofisher). All samples were analysed in duplicate in a 10 μl reaction volume. All qPCR reactions were run at 95 °C for 10 min, 45 cycles of 95 °C for 15 s and 60 °C for 1 min.

Data analysis was done with the QuantStudio Real-Time PCR Software version 1.3 (Applied Biosystems). The difference in cycle threshold (Ct) values were reported as a representative estimate of change in bacterial load between time-points. Lower limit of detection was determined as the Ct from no template controls for each primer/probe set. For all reactions, a Ct value of 40 was considered the threshold for detection.

### Statistical analysis

Data analysis were performed using IBM SPSS v.24 and graphs were prepared by GraphPad Prism v.7. The change in the proportion/number of immune cells, the change in the bacterial loads between time points, and baseline cytokines levels were analysed using a non-parametric approach (Wilcoxon Signed Ranked test or Mann-Witney U test, respectively). The co-primary endpoints for cytokine analysis were impact of sampling on levels of the pro-inflammatory cytokine IL-1α and the chemoattractant IL-8. For better visualization, changes in cytokines were demonstrated using forest plots with log10-transformation of the data, confirmation of normality, and comparison by paired t-test. A p-value < 0.05 was considered significant; in all cases non-parametric analysis was also performed, and gave similar results.

## Supplementary information

Supplementary Information.

## Data Availability

All data generated or analysed in this study will be provided by the corresponding author on reasonable request.
