# Supplementary material for: The impact of cervical cytobrush sampling on cervico-vaginal immune parameters and microbiota relevant to HIV susceptibility
Source: Sci Rep. 2020 May 22;10:8514. doi: 10.1038/s41598-020-65544-6 (PMC7244754; doi:10.1038/s41598-020-65544-6)
Supplement: Supplementary file 1 — Supplementary Information. [file 41598_2020_65544_MOESM1_ESM.pdf]

The impact of cervical cytobrush sampling on cervico-vaginal immune parameters and microbiota relevant to HIV susceptibility

A Mohammadi[1], S Bagherichimeh[1], M.C Perry[2], A Fazel[1], E Tevlin[3], S Huibner[1], W Tharao[3], B Coburn[4,2], R Kaul[1,2]

[1]Department of Medicine, University of Toronto, Toronto, Canada

[2]Department of Immunology, University of Toronto, Toronto, Canada

[3]Women's Health in Women's Hands Community Health Center, Toronto, Canada

[4]Department of Laboratory Medicine and Pathobiology, University of Toronto, Toronto, Canada

Correspondence: Dr. Rupert Kaul and/or Mrs. Avid Mohammadi. Department of Medicine, University of Toronto, Medical Sciences Building #6356, Toronto, Ontario, Canada, M5S 1A8. Tel: (1-416) 978-8607; Fax: (1-416) 978-8765.

SUPPLEMENTARY MATERIALS

Table 1S. Primer and primer sets used for detection and quantification of key bacterial species

Target	Oligo	Sequence	Assay concentration
16S	Forward	TCCTACGGGAGGCAGCAGT	300 nM
	Probe	[FAM]CGTATTACCGCGGCTGCTGGCAC[MGB]	200 nM
	Reverse	GGACTACCAGGGTATCTAATCCTGTT	300 nM
Prevotella spp.	Forward	CCAGCCAAGTAGCGTGCA	300 nM
	Probe	[FAM]AATAAGGACCGGCTAATTCCGTGCCAG[TAM]	200 nM
	Reverse	TGGACCTTCCGTATTACCGC	300 nM
Prevotella bivia	Forward	GGATATTTGGCATCATCAGTGGTATC	300 nM
	Probe	[FAM]TGATTTGGGGGTTGGGCGATTT[BHQ1]	200 nM
	Reverse	ATAACTGATTGGAAGATACGCTACCA	300 nM
Lactobacilli spp.	Forward	CGTGGTTCAGCWTTGAAGGC	800 nM
L. iners	Probe	[HEX]AGGCGATCCAGAACAAGAAGCAG[BHQ1]	100 nM
L. crispatus	Probe	[ROX]AGGCGACAAGGAAGCTCAAGAAC[BHQ2]	100 nM
L. gasseri	Probe	[FAM]AGGTGACCCAGAACAACAAGACG[BHQ1]	50 nM
L. jensenii	Probe	[Cy5]AGGTGACCCAGAACAAGAAAAGGT[BHQ2]	100 nM
	Reverse	CTTCAACTGGCATYAAGAATGGC	800 nM
A. vaginae	Forward	TAGGTCAGGAGTTAAATCTG	500 nM
	Probe	[VIC]CTACCAGACTCAAGCCTGCC	300nM
	Reverse	TCATGGCCCAGAAGACCGCC	500 nM
G. vaginalis	Forward	GCGGGCTAGAGTGCA	800 nM
	Probe	[JUN]CTTCTCAGCGTCAGTAACAGC	300 nM
	Reverse	ACCCGTGGAATGGGCC	800 nM
	Forward	GATGCCAACAGTATCCGTCCG	500 nM
	Probe	[FAM]ACAGACTTACCGAACCGCCT	150 nM
	Reverse	CCTCTCCGACACTCAAGTTCGA	500 nM

SUPPLEMENTARY FIGURE 1. Gating strategy and representative plots for endocervical cells.

a1) Gating strategy for endocervical CD4+ T cell and Th17 cells

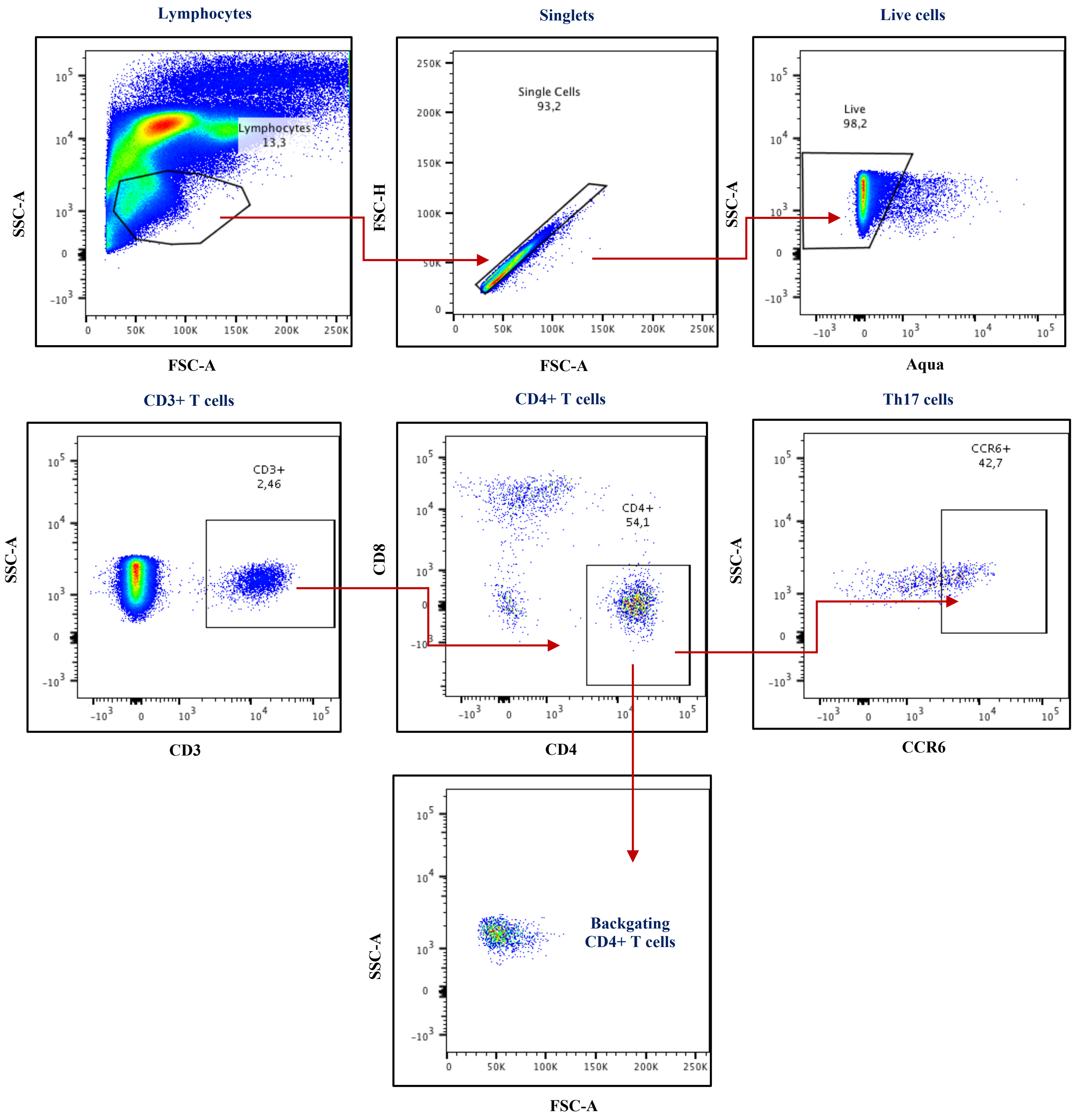

a2) Gating strategy for endocervical CCR5+ CD4+ T cells, CD69+ CD4+ T cells, activated CD4+ T cells and memory CD4+ T cells

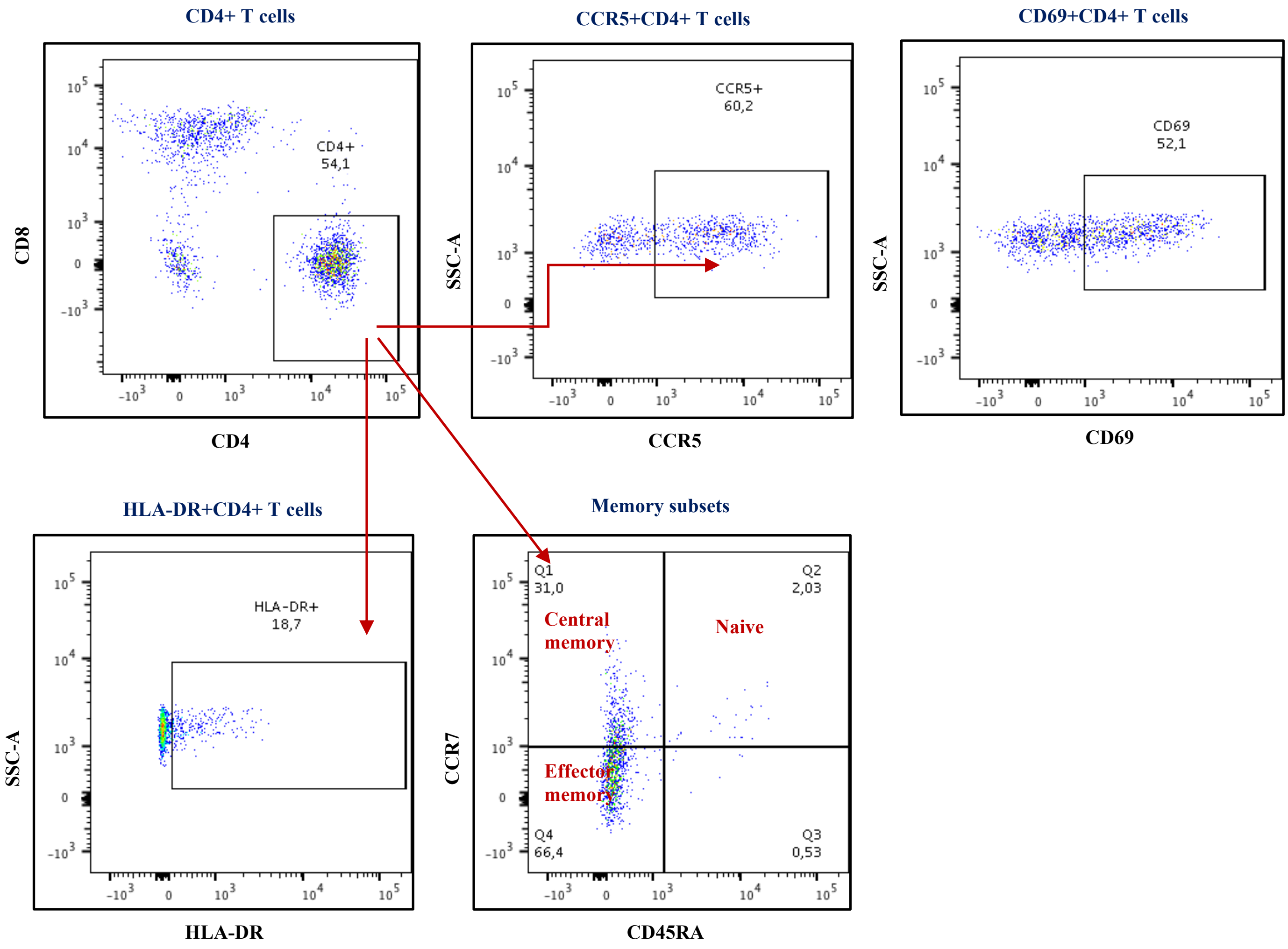

b) Gating startegy and backgating for endocervical activated neutrophils

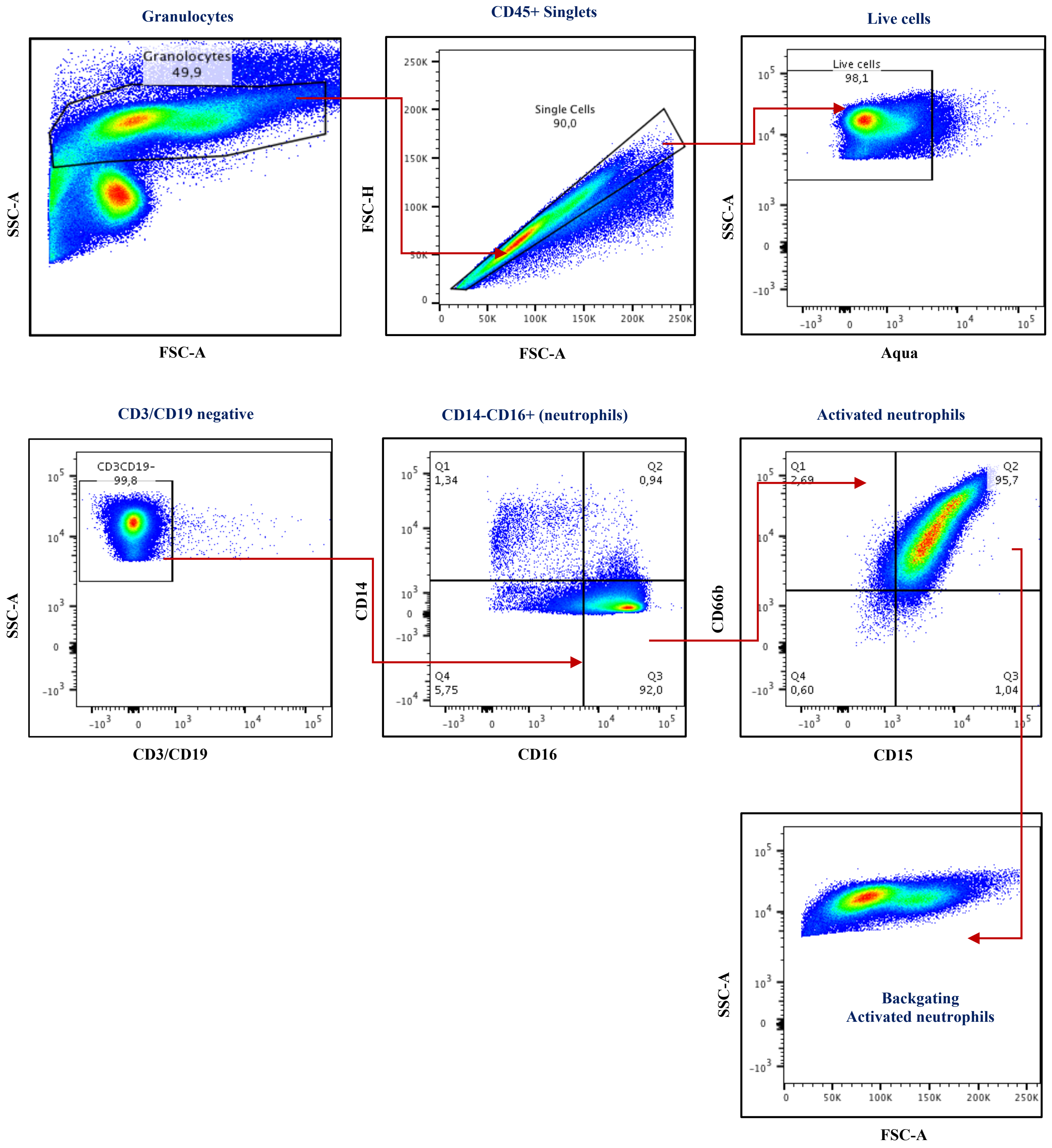

c) Gating strategy for endocervical monocytes/macrophages

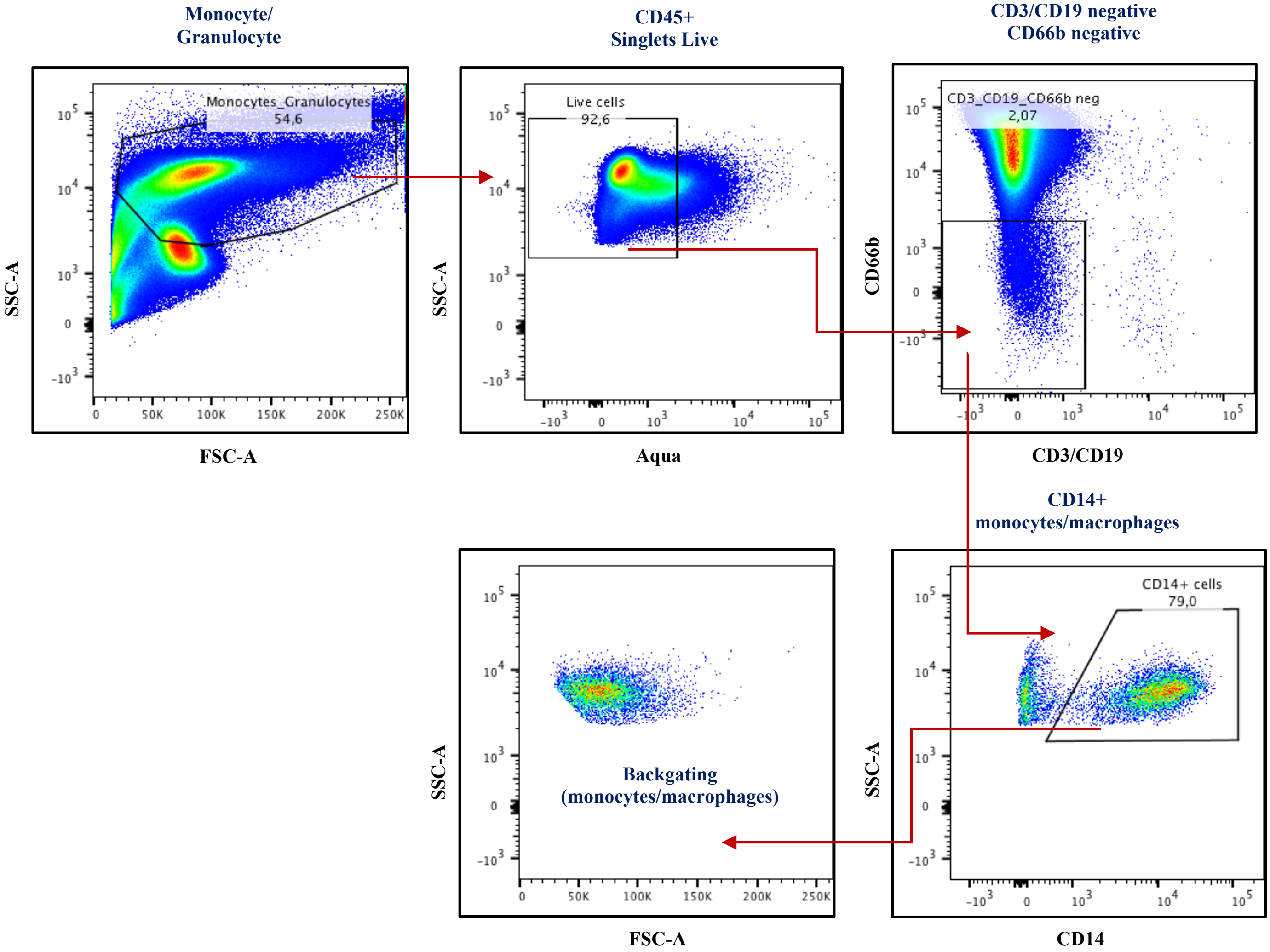

d) Gating strategy for endocervical CD14+ Dendritic cells and CD14 negative Dendritic cells

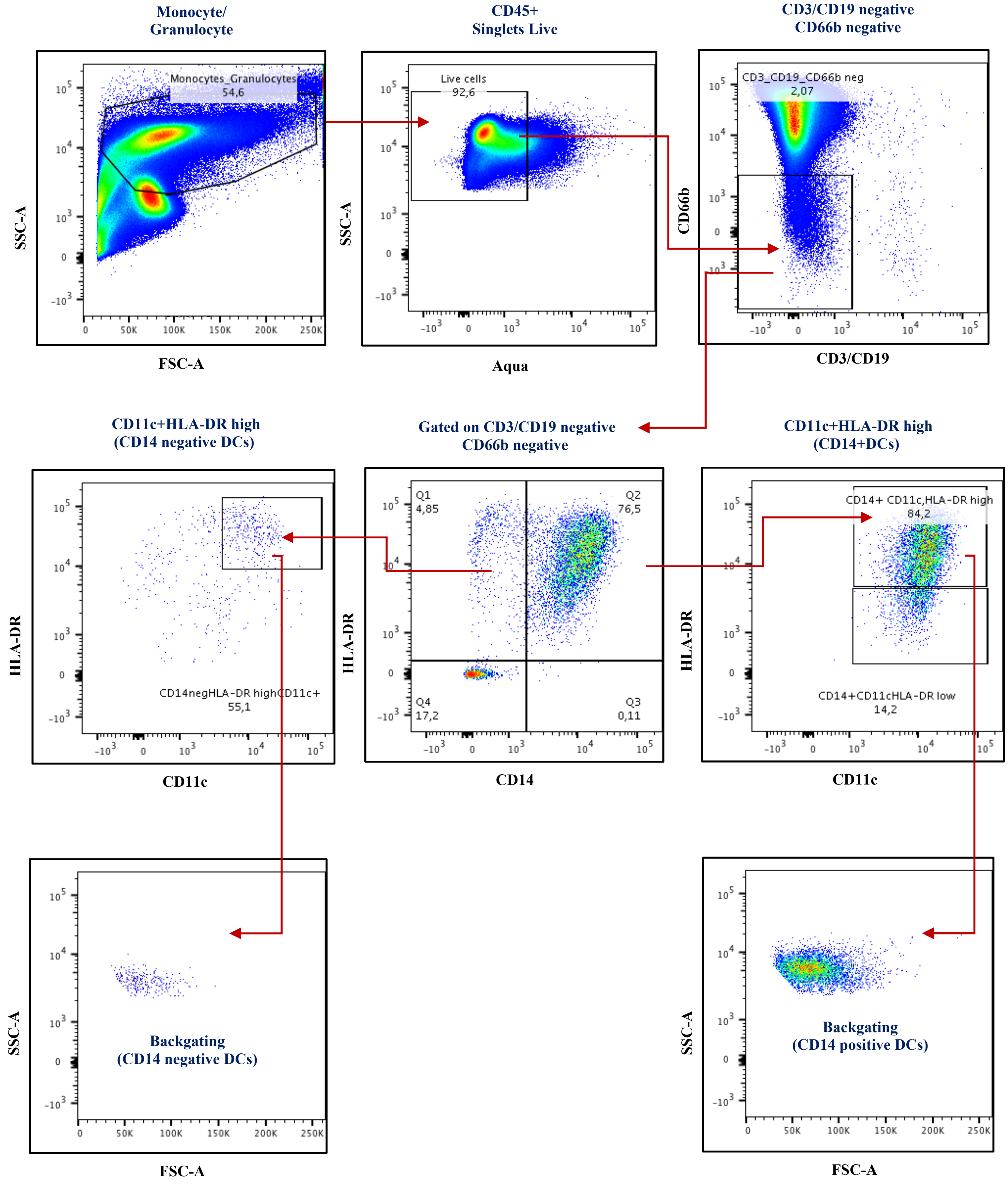

Supplementary Figure 1. Gating strategy and representative plots for endocervical cells. (**a1)** Cells were gated on lymphocytes, singlets, live, CD3+ cells, CD4+ cells, CCR6+ CD4+ T cells (Th17). Backgating is shown to demonstrate the validity of gating strategy (**a2)** Cells were gated on CD4+ cells, CCR5+ CD4+ T cells, CD69+ CD4+ T cells, HLA-DR+CD4+ T cells (activated CD4+ T cells), CD4+ memory subsets (Central memory: CCR7-CD45RA+; effector memory: CCR7-CD45RA- and naive cells: CCR7+CD45RA+) **(b)** Cells were gated on granulocytes, CD45+ cells, singlets, live cells, CD3/CD19 negative cells, neutrophils (CD14 negative CD16 positive), activated neutrophils (CD15+ cells expressing CD66b+). Backgating is shown to demonstrate the validity of gating strategy **(c)** Cells were gated on monocytes, CD45+ cells, singlets, CD3/CD19 negative, CD66b negative cells, CD14+ cells. Backgating is shown to demonstrate the validity of gating strategy **(d)** Cells were gated on monocytes, CD45+ cells, singlets, CD3/CD19 negative, CD66b negative cells, based on CD14 and HLA-DR expressions two subsets of DCs: CD14+ DCs (CD11c+HLA-Dr highCD14+), CD14- DCs (CD11c+ HLA-DR highCD14-). Backgating is shown to demonstrate the validity of gating strategy.

SUPPLEMENTARY FIGURE 2. The level of cervico-vaginal cytokine concentrations at baseline.

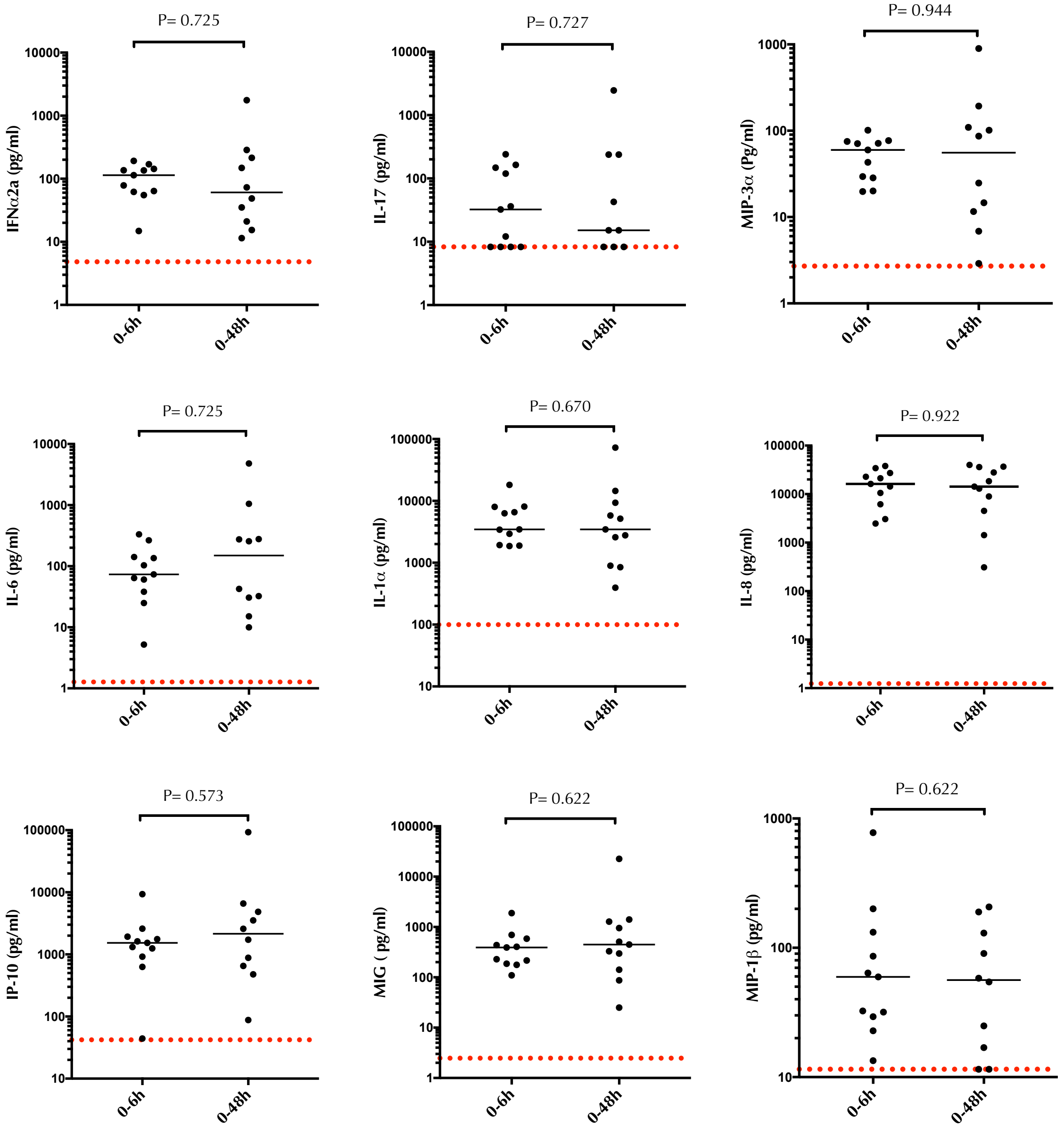

Supplementary Figure 2. The level of cervico-vaginal cytokine concentrations at baseline. The level of cervico-vaginal cytokine concentrations is compared between 0-6hrs and 0-48hrs group. The dotted line indicates the LLOD. The LLODs were as follow: IFNα2a= 4.82pg/ml; IL-17= 8.3pg/ml; MIP-3α= 2.7pg/ml; IL-6= 1.27pg/ml; IL-1α= 99.6pg/ml; IL-8= 1.24pg/ml; MIG= 2.47pg/ml; IP-10= 42.1pg/ml; MIP-1β= 11.5pg/ml. Statistical comparisons were performed using Mann-Witney U test.
